# Trachoma

**DOI:** 10.1016/S0140-6736(25)00551-3

**Published:** 2025-05-24

**Authors:** Esmael Habtamu, Emma M. Harding-Esch, Katie Greenland, Teyil Wamyil-Mshelia, Sandra L. Talero, Sailesh Kumar Mishra, Thomas Lietman, Anthony W. Solomon, Matthew J. Burton

**Affiliations:** 1 Clinical Research Department, London School of Hygiene & Tropical Medicine, London, UK; 2 Eyu-Ethiopia, Bahir Dar, Ethiopia; 3 Department of Ophthalmology, College of Medicine and Health Science, Bahir Dar University, Bahir Dar Ethiopia; 4 Department of Disease Control, London School of Hygiene & Tropical Medicine,London, UK; 5 Sightsavers Country Office, Abuja, Nigeria; 6 Neglected, Tropical and Vector-Borne Diseases Unit, Communicable Diseases and Environmental Determinants of Health Department, Pan American Health Organization,Washington DC, USA; 7 Nepal Netra Jyoti Sangh,Kathmandu, Nepal; 8 Francis I Proctor Foundation, University of California, San Francisco, California, USA; 9 Global Neglected Tropical Diseases Programme, World Health Organization, Geneva, Switzerland; 10 National Institute for Health Research Biomedical Research Centre for Ophthalmology at Moorfields Eye Hospital NHS Foundation Trust and UCL Institute of Ophthalmology, London, United Kingdom

## Abstract

Trachoma, the leading infectious cause of blindness worldwide, is one of several neglected tropical diseases targeted for elimination by 2030. The disease starts in childhood with repeated episodes of conjunctival *Chlamydia trachomatis* infection. This is associated with recurrent conjunctivitis (active trachoma), which if left untreated, leads to cicatricial trachoma characterised by scarring of the conjunctiva, and potentially in-turned eyelashes (trachomatous trichiasis) in later life. Trachoma mainly affects the poorest and most rural communities; these populations typically have limited access to water and hygiene facilities. Blinding complications are most commonly seen in females, who in many cultures act as caregivers for infected children from a young age. To eliminate trachoma as a public health problem, programmes implement a package of interventions known as “SAFE”: **S**urgery to treat trachomatous trichiasis, **A**ntibiotic mass drug administration to treat infection, **F**acial cleanliness, and **E**nvironmental improvement to limit transmission. The SAFE strategy has brought considerable success in the last two decades. As of December 2024, 21 countries have eliminated the disease, while several others are on track to eliminate it soon. However, persistent and recrudescent active trachoma in some populations may challenge the success of the 2030 global elimination target. In such settings, novel, or more intensive approaches must be promptly developed, tested, and scaled-up to accelerate elimination.

## Introduction

Trachoma is a neglected tropical disease, and the leading infectious cause of blindness worldwide.^1,2^ Precipitated by recurrent episodes of infection with ocular strains of the bacterium *Chlamydia trachomatis* (*Ct*), trachoma begins in childhood as a chronic kerato-conjunctivitis known as active trachoma. This results in immuno-pathologically mediated conjunctival scarring (TS), which causes eyelid in-turning (entropion) with eyelashes scratching the eye (trachomatous trichiasis, TT). TT can cause painful corneal abrasions and ulceration. Resolution of ulcers is accompanied by corneal opacification, leading to vision impairment.

Trachoma has major personal, social and economic consequences for affected individuals, families and communities.^3^ TT results in significant morbidity, functional and physical impairment, social withdrawal and exclusion, and reduced ability to work and earn an income, adversely affecting vision- and health-related quality of life, even before vision impairment develops.^4–7^ The global productivity loss from trachoma was estimated in 2003 at US$8 billion annually.^8^

In the 25 years, the global effort to eliminate trachoma as a public health problem has resulted in dramatic declines in the global burden of the disease.^9^ However, despite sustained interventions, it still affects the poorest communities, predominantly in Africa.^9–12^ In this seminar, we provide a distillation of the key clinical and public health features of trachoma.

## Clinical Features and Disease Mechanisms

### Clinical Features and Course

**Figure 1:**
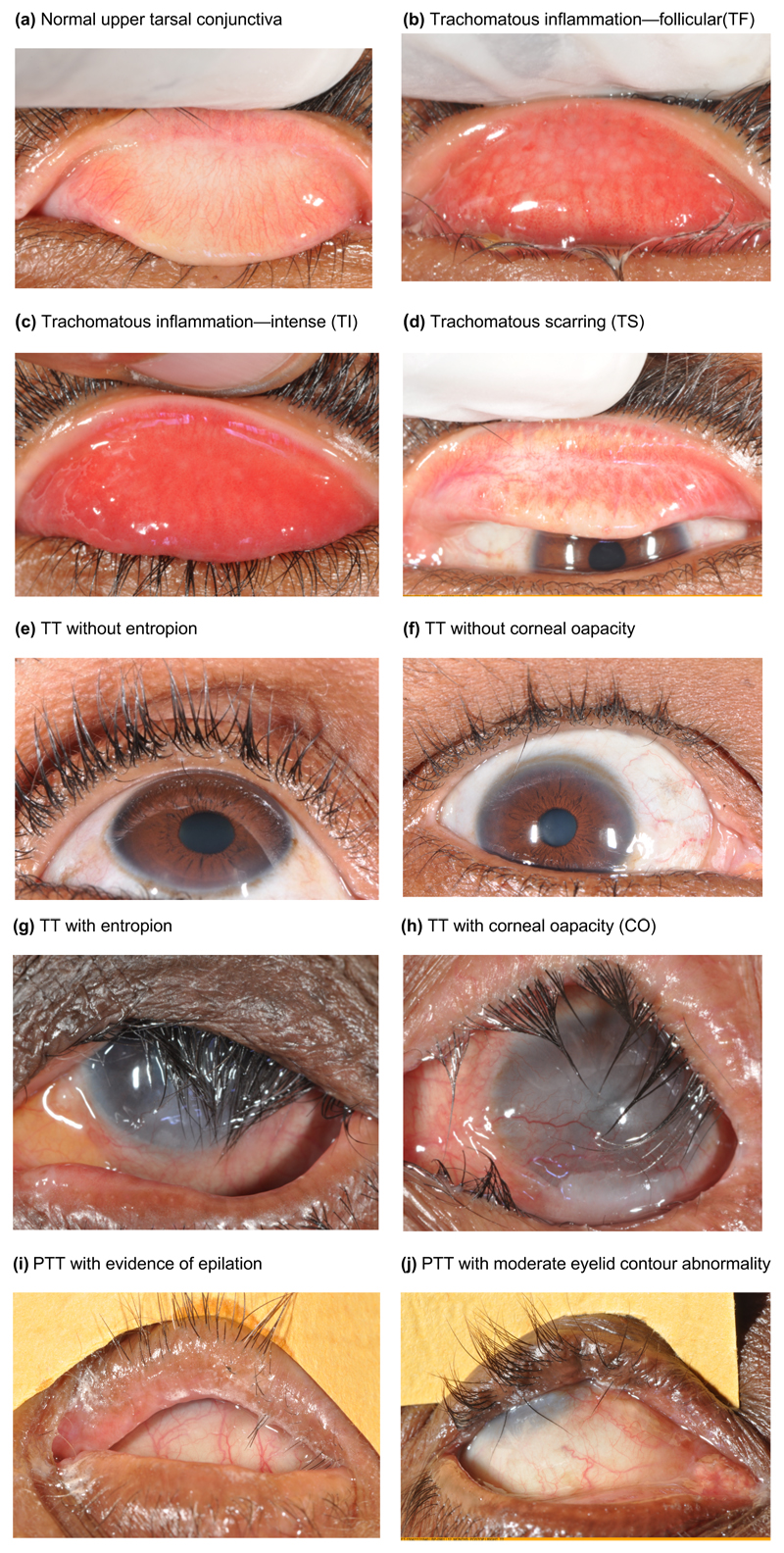

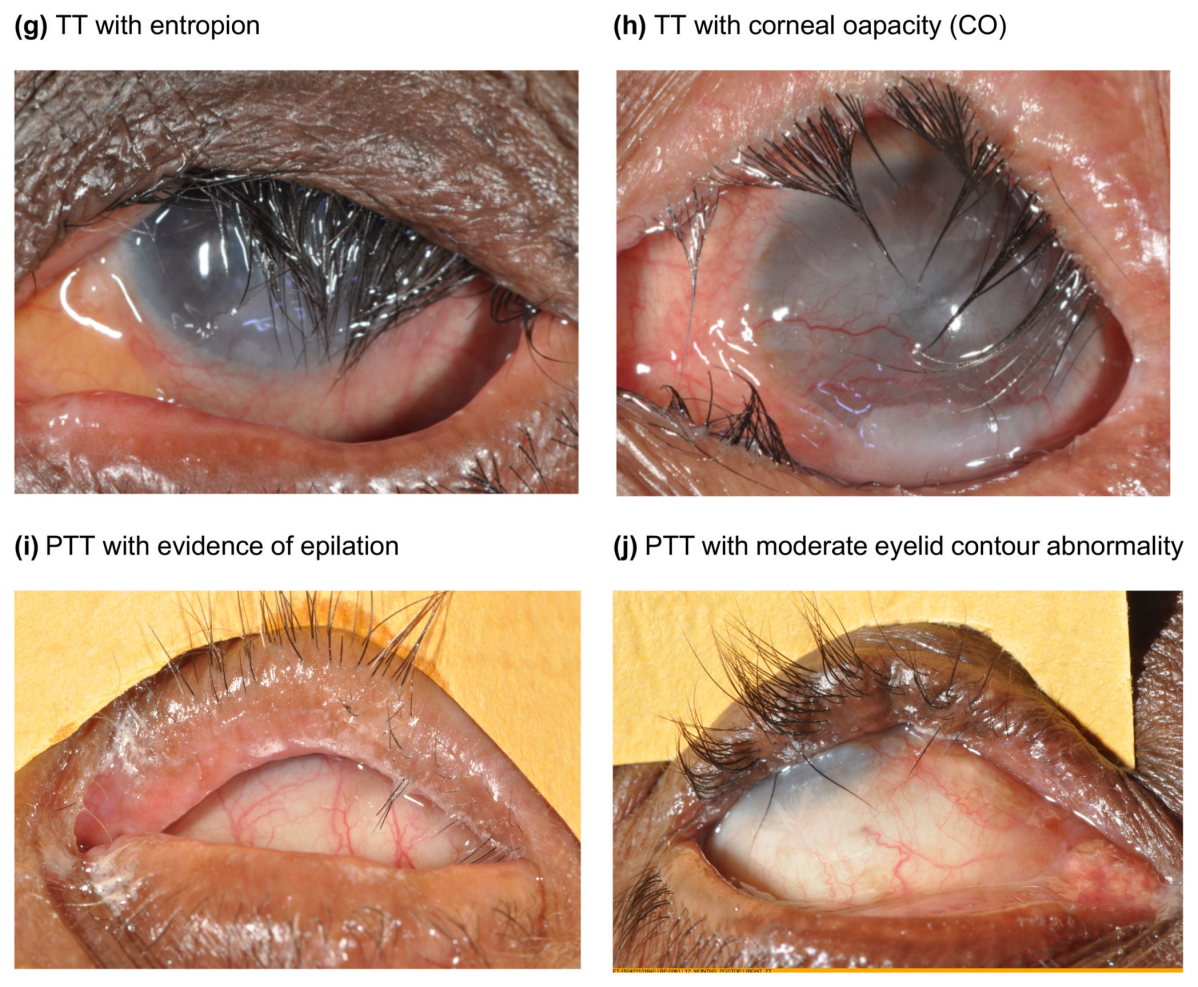
Clinical features of trachoma and the WHO simplified grading system (as amended in 2020)^14^. Note: A normal upper tarsal conjunctiva is shown for comparison as panel (a). Panels (b) to (h) illustrate the clinical features of trachoma along with the WHO simplified grading system as follows: Panel (b) Trachomatous inflammation—follicular (TF), the presence of five or more follicles, each at least 0.5mm in diameter, in the central part of the upper tarsal conjunctiva (equivalent to Zone 2 plus Zone 3 in Figure 4). Panel (c) Trachomatous inflammation—intense (TI), pronounced inflammatory thickening of the upper tarsal conjunctiva that obscures more than half of the deep normal vessels. Panel (d) Trachomatous scarring (TS), the presence of easily visible scarring in the upper tarsal conjunctiva. Panels (e), (f), (g), (h) Trachomatous Trichiasis (TT), at least one eyelash from the upper eyelid touches the eyeball, or evidence of recent epilation of in-turned eyelashes from the upper eyelid. Panel (h) Corneal Opacity (CO), easily visible corneal opacity that is so dense that at least part of the pupil margin is blurred when viewed through the opacity. Panels (i) and (j) show PTT= Postoperative TT (TT after surgery).

The ocular strains of *Ct* cause a follicular conjunctivitis in children. While the follicles can be found throughout the palpebral and bulbar conjunctiva, they typically form a characteristic pattern in the upper eyelid conjunctiva: off-white, elevated, round, evenly spaced germinal centres. This can meet the definition of the sign trachomatous inflammation—follicular (TF, Figure 1b). TF can be associated with redness (hyperaemia) and inflammation (papillary reaction), which if florid, qualifies as trachomatous inflammation—intense (TI, Figure 1c), although these features are less specific for trachoma than the follicular pattern. The presence of TF and/or TI is known as “active trachoma”. While a single infection episode can clear within months, repeated infections may lead to conjunctival scarring. Several patterns of scars, trachomatous scarring (TS, Figure 1d) can be seen, including linear scars connecting necrotic follicles in a reticular pattern. Far less commonly, a horizontal linear scar forms in the upper eyelid, between the marginal and superior blood vessels (Arlt’s line), which has been called pathognomonic for trachoma. Progressive conjunctival scarring can lead to entropion and trachomatous trichiasis (TT, Figure 1g). Corneal damage results from pannus (fibrovascular change) and secondary infectious ulcers, leading to opacity (corneal opacity, (CO), Figure 1h) and vision impairment. When they occur, the follicular conjunctivitis and cicatricial changes including entropion, TT, and CO are typically bilateral, ultimately causing blindness.^13^

### Chlamydia trachomatis

**Figure 2:**
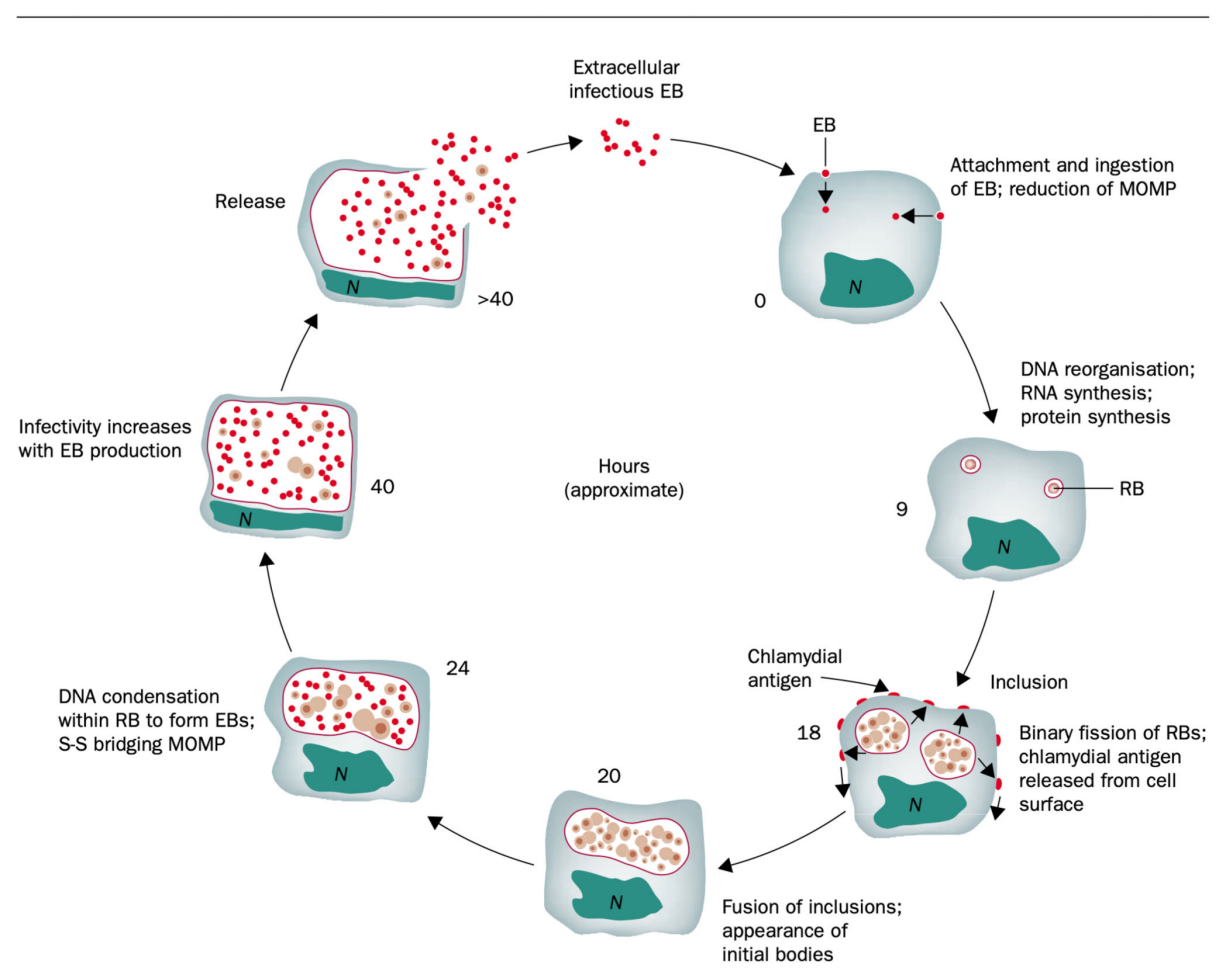
Life cycle of *Chlamydia trachomatis*. Adapted from Mabey *et al*., 2003.^19^

*Ct* is a bacterium that reproduces inside host cells. The intracellular replicative form is known as a reticulate body. It inhabits a membrane-bound vacuole called an inclusion. Towards the end of the replicative cycle (Figure 2), elementary bodies (EBs) are formed, which have a rigid cell wall to prolong survival in the intercellular milieu. EBs are non-motile, but following host cell lysis, they can attach and enter new host cells in the same or a different host, initiating a further cycle of replication.

*Ct* is classified into different serovars based on variations in its major outer membrane protein.^15,16^ Serovars A, B, Ba, and C are primarily associated with trachoma. Serovars D–K are mostly associated with urogenital tract infection, and serovars L1–3 cause the more invasive sexually transmitted disease, lymphogranuloma venereum. These different *Ct* lineages have emerged relatively recently in human history.^17^

The genome of *Ct* includes a 1,042,519-base pair chromosome and a 7493-base pair plasmid.^18^ A thorough understanding of the genome and proteome of *Ct* may be crucial for developing effective interventions to eliminate trachoma.

### Relationship Between Infection and Active Trachoma

In cross-sectional studies of trachoma-endemic populations, there is often a marked mismatch at the individual level between the presence of active trachoma and the detection of *Ct* by polymerase chain reaction (PCR) or nucleic acid amplification tests (NAATs).^20,21^ In contrast, at the population level, active trachoma and detectable *Ct* are less tightly correlated. The individual mismatch reflects the different time courses of infection and disease episodes. The few cohort studies on the relative time courses indicate that individual episodes of infection vary in duration from a few days to a few weeks.^22^ In contrast, particularly in young children, inflammatory episodes of active disease may persist for several months in the absence of reinfection and well after *Ct* is no longer detectable. This has important implications for trachoma control, as the presence of active trachoma in an individual is not a consistently reliable indicator of current *Ct* infection.

### Disease Mechanisms

Following introduction of *Ct* to the ocular surface, an active, replicating infection can become established in the conjunctival epithelium. Active trachoma is characterised at the tissue level by a mixed inflammatory cell infiltrate (lymphocytes, neutrophils and macrophages), dotted with lymphoid follicles, composed mostly of B cells. At the molecular level, active trachoma is associated with marked upregulation in pro-inflammatory cytokines and chemokines, as well as extracellular matrix modifiers. Resolution of *Ct* infection is believed to involve a TH1 cell mediated immune response, involving interferon-γ.^23^ Additionally, active trachoma and *Ct* infection are associated with T_H_17 responses, with some indication that this may promote inflammation and scarring.

The prevalence of the scarring complications of trachoma accrues with increasing age. Longitudinal studies with multiple clinical observation timepoints have consistently found a strong association between the presence of repeated episodes of conjunctival papillary inflammation (TI) and incident and/or progressive conjunctival scarring.^24–28^ The association between detectable episodes of *Ct* and scarring is weaker. It is possible that there is intrinsic variation in how individuals respond to *Ct* exposure, with some people developing a more marked chronic inflammatory response, leading to more extensive tissue damage and scarring on resolution. Individuals with more frequent exposure to reinfection are probably at greater risk of developing scarring.^29^

Inflamed conjunctiva with evolving scarring exhibits increased expression of several matrix metalloproteinases (MMP,7, MMP9 and MMP12), growth factors and pro-inflammatory genes (*IL1β, IL-17A, CXCL5* and *S100A7*), which may promote scarring development.^25^

## Epidemiology and Transmission

**Figure 3:**
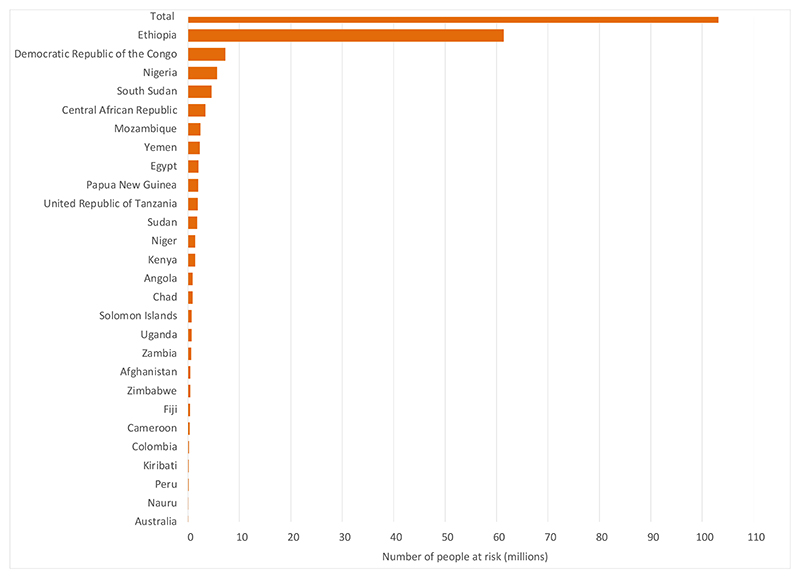
Estimates of the population at risk for trachoma by country (WHO data^30^)

In April 2024, trachoma was known to be a public health problem in 39 countries in Africa, Asia, Central and South America, Australia, and the Middle East, with 103·2 million people at risk of developing blindness due to trachoma unless interventions are successfully implemented, Figure 3.^30^ About 90% (93·1 million) of the global burden of trachoma is found in WHO’s African Region.^30^ Ethiopia is the most affected country, accounting for about 59% of the global burden, with ~61·4 million people living in trachoma-endemic districts in April 2024.^30^ The global burden of TT was estimated at 1·5 million people in 1,726 districts.^30^

*Ct* likely leaves the ocular surface in tear fluid and nasal secretions.^31^ Evidence then points to direct onward transmission of *Ct* via multiple routes, including on skin, fomites and eye-seeking flies. Studies mapping the presence of *Ct* using PCR tests of surface swabs in an endemic environment found detectable *Ct* DNA on faces, hands, fabrics and flies.^32^

Active trachoma and conjunctival *Ct* infection consistently exhibit marked geospatial clustering,^33,34^ suggesting localised transmission is dominant, within households and parts of communities. Crowded living conditions are associated with trachoma, perhaps indicating increased opportunities for transmission events.^35^ Other markers of poverty, such as limited access to water and sanitation, are also strongly associated with trachoma.^9,36,37^

Unclean faces, particularly characterised by peri-ocular secretions, are frequently associated with the individual-level presence of active trachoma and *Ct* and may be an important part of the transmission pathway.^38–40^ Limited access to water for washing faces, hands and clothes makes it harder to keep these surfaces clean, in order to limit transmission.

The primary eye-seeking fly reported in many trachoma-endemic environments is *Musca sorbens*.^41^ This preferentially breeds in human faeces. Environments with limited access to sanitation and greater faecal contamination have more breeding sites to support a larger fly population.^42^ Female *M. sorbens* are particularly attracted to the eyes of children with active trachoma and ocular discharge.^10,39,42,43^

The signs of active trachoma and ocular *Ct* infection are most prevalent in pre-school age children. At this age, boys and girls tend to be equally affected.^20^ In contrast, the scarring sequelae of trachoma, which appear in older age groups, invariably affect women more frequently than men. This gender difference is usually attributed to a greater lifetime exposure to ocular *Ct* infections among women, who are often the primary caregivers of young children, and therefore likely to be more frequently exposed.^44^

## Diagnosis

### Clinical Grading

In programmatic and epidemiological investigations, trachoma is typically diagnosed through signs detected by trained clinical observers using a torch and a magnification loupe.^45^ The upper eyelid is examined for entropion and TT, including evidence of epilation. Active trachoma (TF, TI) and conjunctival scarring are assessed by everting the upper eyelid. Population-based surveys typically report the prevalence of TF in children aged 1–9 years, while the prevalence of TT is reported in people aged 15 years and older.

**Figure 4:**
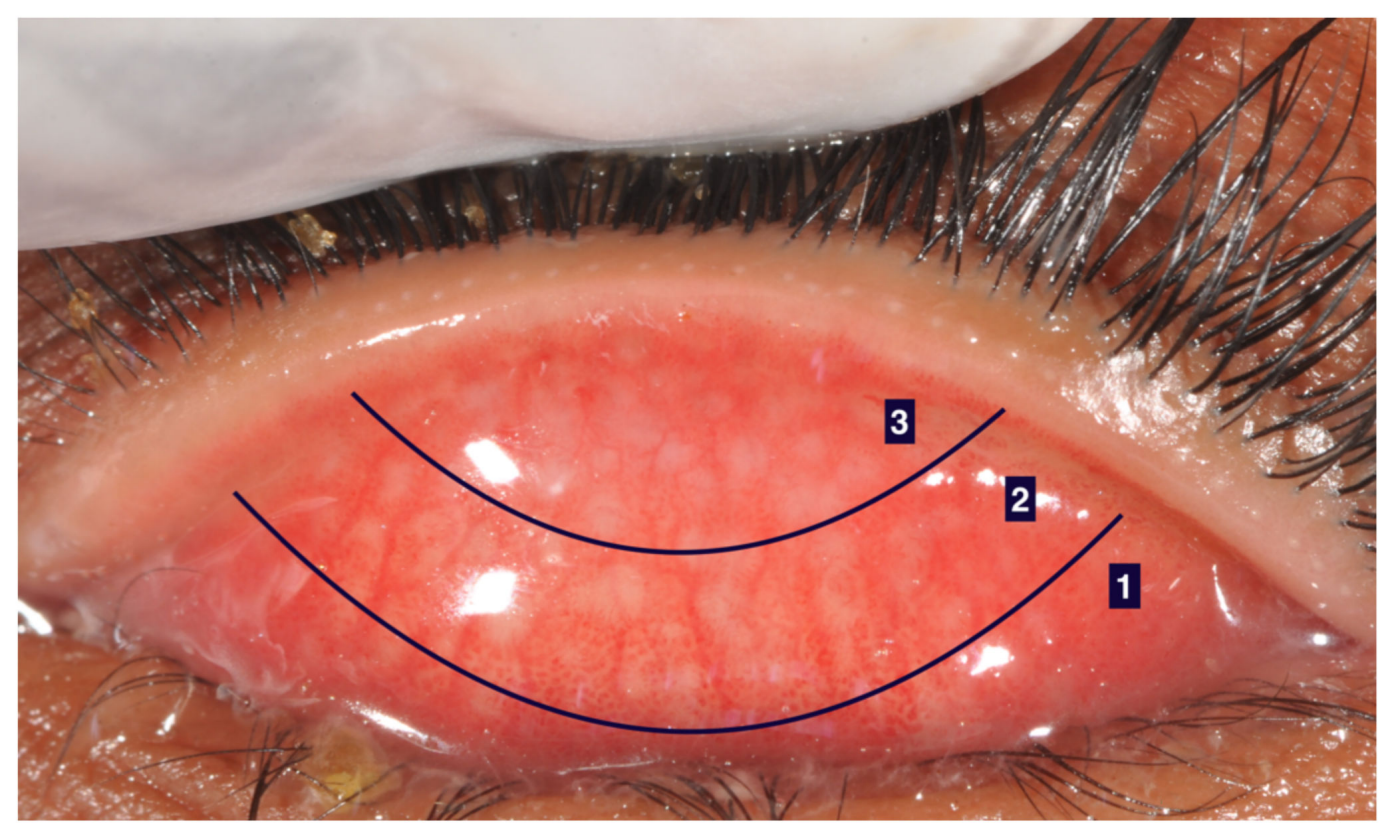
An everted upper eyelid, showing the gradable sections in the Follicles-Papillae-Cicatricae (FPC) and simplified grading systems

WHO has two grading systems for trachoma: the more detailed Follicles-Papillae-Cicatricae (FPC) grading and the simplified trachoma grading.^14,46–48^ The FPC system was developed to grade severity of (1) trachomatous follicles, (2) papillary inflammation, (3) conjunctival scarring, (4) TT and/or entropion and (5) corneal scarring, Table 1. This grading system has been useful for assessing variation in disease severity, particularly in research studies. However, it is more complex than needed for programmatic decision-making, for which examinations are usually performed by non-specialists in field settings. As a result, the simplified grading system was developed for programmatic use. This defines the presence or absence of key clinical features, (see Figure 1 footnote).^14^ The gradable sections in FPC and the simplified grading system are shown in Figure 4.

The numbered zones refer to those in the FPC Grading System. Zones 3 and 2 are examined for trachomatous inflammation---follicular when using the WHO simplified system.^47^

#### Laboratory

Trachoma is a disease caused by an infection; not the infection itself. However, laboratory diagnosis of current or past ocular *Ct* infection is increasingly finding programmatic application in global trachoma elimination efforts.^49–51^

The gold standard assays for current *Ct* infection are NAATs.^45^ These detect the presence of *Ct* DNA or RNA with high sensitivity and specificity. NAATs are performed on conjunctival swabs, which can be collected in a standardised way to obtain approximately consistent volumes of cellular and extracellular material.^52^ Non-commercial (“home-brew”) quantitative PCR assays are also available.^52–54^ Viability PCR, which uses propidium monoazide to prevent amplification of DNA from membrane-impaired bacteria, has recently been used to investigate ocular *Ct* transmission routes.^55^

Beyond NAATs, there are a variety of older techniques to detect current infection, including microscopy-based tests, enzyme-linked immunosorbent assays (ELISA) to detect antigen, and chlamydial culture.^45^ They are now rarely used. Culture is highly specific and has the singular advantage of facilitating the assessment of antimicrobial susceptibility,^56^ but is labour-intensive, requires specialised cell lines and incubation conditions, and is poorly reproducible. *Ct* antibiotic resistance evaluation is increasingly undertaken genotypically.^57^

There is a growing appreciation of the shortcomings of using TF prevalence alone to guide decision-making on antibiotic mass drug administration (MDA), a cornerstone of trachoma elimination programmes. Seroprevalence (or seroconversion rate) in young children, based on antibodies to the *Ct* antigen Pgp3, is emerging as a useful complementary index.^58,59^

## Prevention & Management

### Overview

The SAFE strategy, introduced in 1993, is a comprehensive approach to trachoma control, focusing on **S**urgery for TT, **A**ntibiotics to clear Ct infection, and **F**acial cleanliness and **E**nvironmental improvement (water and sanitation) to limit chlamydial transmission.^60^ Current prevention efforts align with this strategy, aiming to limit Ct transmission and prevent blindness. Although no chlamydia vaccine is commercially available, a vaccine based on the recombinant protein subunit CTH522 has now completed two Phase I trials.^61,62^ These trials assessed the safety and immunogenicity with different adjuvants. CTH522 adjuvanted with CAF01 liposomes induced stronger T-cell and antibody responses than when combined with aluminium hydroxide.^61^ Various regimens of CTH522 with CAF01 or CAF09b adjuvants were safe across intramuscular, intradermal, and ocular administration routes, with the latter two enhancing mucosal immunity.^62^

### Surgery

Surgery is provided to people with TT to reduce their risk of sight loss. The aim of surgery is to reposition the eyelashes, limiting the damage they cause from abrading the cornea. Patients also benefit from a marked reduction in pain and may experience some improvement in vision.^63^ The current WHO recommendation is that surgical management should be offered to all patients having TT with eyelid entropion.^64^ Many different surgical procedures have been described to correct TT.^65–68^ These range from eyelid margin splinting to excision of the tarsus. Some surgical procedures address the eyelid margin and the anterior lamella, while others lengthen or rotate the tarsal conjunctiva.^65^ The two most commonly used surgical procedures are the Bilamellar Tarsal Rotation (BLTR) and Posterior Lamellar Tarsal Rotation (PLTR) procedures.^64^ WHO currently recommends new surgeons are trained in the PLTR procedure^69^ following the results of a clinical trial that found PLTR to be superior to BLTR in reducing postoperative TT (PTT), the presence of TT after surgery.^70,71^

Not all TT requires surgery. WHO recommends that patients with non-entropic TT, TT in which eyelashes do not touch the cornea in the primary position of gaze, and those declining surgery after appropriate counselling can be offered high-quality eyelash epilation. Epilation is commonly practised in many trachoma-endemic countries.^65,72–74^ Studies indicate that epilation can be used as an alternative to surgery in people with minor (≤5 eyelashes touching the eye or evidence of epilation in <1/3^rd^ of eyelid margin) unoperated and postoperative TT.^74–76^

### Antibiotic

*Ct* is susceptible to several antimicrobial classes, including tetracyclines, sulfonamides, beta-lactams, and macrolides.^77^ Oral azithromycin has particular advantages in its safety profile and long tissue half-life. Following studies indicating that a single dose of oral azithromycin (20 mg/kg up to 1 gram) was comparable to 6 weeks of topical tetracycline in eliminating ocular *Ct* infection from individuals,^78–80^ and that three doses of oral azithromycin MDA to those aged ≥1 year were at least as effective as the longer course of topical tetracycline,^81^ WHO recommended single-dose annual azithromycin MDA.^82^ The number of MDA rounds planned depends on the prevalence of TF in 1–9-year-olds: ≥30% warrants 5 annual MDA rounds before re-survey; ≥10–29·9% warrants 3 annual rounds before re-survey; and ≥5–9·9% warrants one round before re-survey.^82,83^ Single-dose annual azithromycin MDA was proven to dramatically reduce infection.^84,85^

Mathematical models predict that more severely affected communities might require more-frequent-than-annual distributions to achieve success.^86,87^ Biannual and quarterly MDA have reduced infection rapidly, and in some cases completely eliminated infection from even the most hyper-endemic populations.^88–93^ Distributing antibiotics to entire communities has raised concern over selection of antibiotic-resistant bacteria. While azithromycin resistance has not been detected in conjunctival *Ct* itself, selection for resistance has been found in other bacterial pathogens, such as *Streptococcus pneumoniae*, in populations post-MDA.^94,95^ Strategies to mitigate selection of resistance and overall antibiotic exposure may include restricting distribution to the younger age-groups thought to be key for *Ct* transmission.^96,97^

### Facial Cleanliness and Environmental Improvement

Facial cleanliness (‘F’) interventions aim to reduce transmission of *Ct* by removing ocular and nasal discharge from faces. This can be achieved through promotion of regular face washing with soap and complementary practices, such as handwashing and bathing with soap, all of which are facilitated by consistent access to water. Environmental improvement (‘E’) strategies target (i) access to clean water to facilitate “F”, and (ii) control of *M. sorbens* populations directly through insecticidal, trapping or repellent measures,^98,99^ and indirectly by reducing fly breeding sites through sanitation interventions that promote access to and exclusive use of latrines.

Associations between active trachoma, *Ct* infection, water and sanitation access, and periocular fly density have been established.^36,37,41^ Moreover, it is biologically plausible that face washing helps to suppress onward transmission by reducing the presence of infectious oculo-nasal discharge on the face. However, definitively isolating the impact of individual F and E interventions remains challenging due to methodological limitations, heterogeneity in intervention design and implementation, and variations in water access, sanitation coverage, and outcome measurement.^100–107^ Modelling of cross-sectional data suggests that improved access to water is associated with lower active trachoma prevalence at lower coverage levels than those required for sanitation.^107^ This highlights the importance of shifting households onto the ‘water use plateau’ to increase water availability for face washing.^108^ Integrating hygiene promotion into water and sanitation systems strengthening could maximise resource utilisation. Estimating with greater precision the effectiveness of separate F and E interventions would be helpful, particularly for long-term control of trachoma in districts affected by persistent or recrudescent active trachoma. This will require careful consideration of appropriate metrics to measure effectiveness outcomes.^109^

Irrespective of their effect on trachoma transmission, equitable climate-resilient water and sanitation systems along with promotions to encourage sustained uptake of hygiene behaviours through locally tailored, theory- and evidence-based interventions are essential.^110^ Continued investment in and monitoring of these efforts will contribute to health and equity goals that extend beyond trachoma elimination.^111^

### Surveys

To determine where SAFE interventions are needed and for how long, data are needed at the EU (evaluation unit) level. WHO defines an EU, for trachoma elimination purposes, as “the normal administrative unit for health care management, consisting of a population unit between 100,000–250,000 persons”.^82^ Baseline surveys are conducted to determine whether trachoma is a public health problem and if interventions are needed. If TF <5%, no trachoma-specific AFE interventions are needed. If the prevalence of TT unknown to the health system (excluding individuals with post-surgical TT, individuals with TT who have refused surgery for it, and individuals with TT who have a surgical date set in the future) is ≥0·2%, public health level-actions such as active case finding and outreach services are recommended, whereas if the prevalence is <0·2%, remaining and incident TT cases should be managed within routine eyecare services.^83,112^

From 2012–2016, the Global Trachoma Mapping Project (GTMP) conducted baseline surveys in all 1,546 accessible suspected trachoma-endemic districts worldwide (905 EUs), across 29 countries, examining over 2·6 million people, using a standardised survey methodology that conformed to WHO recommendations, and ensuring quality assurance and quality control at every step of the process.^113,114^ Following the GTMP, Tropical Data (TD) was established to support baseline surveys in newly accessible or suspected trachoma-endemic districts, as well as to assist health ministries to conduct impact and surveillance surveys, building on the methods used by the GTMP.^115,116^ WHO recommends that impact surveys be conducted 6–12 months after the last planned MDA round to determine if the TF elimination prevalence threshold has been met or if further interventions are needed, and that surveillance surveys be conducted at least 2 years after an impact survey has returned a TF prevalence <5% to determine whether TF prevalence has remained below 5% in the absence of ongoing MDA.^117^ TD also supports health ministries to conduct TT-only surveys in certain epidemiological contexts.^118^ As of October 2024, TD had supported surveys in over 3800 EUs across 52 countries, covering the examination of over 12·4 million people.

## Elimination As A Public Health Problem

### Validation Process

There are three criteria for elimination of trachoma as a public health problem: (1) a prevalence of TT “unknown to the health system” <0·2% in those aged ≥15 years, and (2) a prevalence of TF <5% in 1–9-year-olds sustained for at least two years in the absence of ongoing antibiotic MDA, in each formerly-endemic district; plus (3) a system to identify and manage incident cases of TT.^119^

WHO has established a process for validating a Member State’s claim that it meets these three criteria.^119^ A dossier should be prepared and submitted to WHO, which convenes an ad-hoc, independent dossier review group to assess the presented evidence. In the event that the dossier review group recommends that the claim be validated, WHO reports the Member State’s achievement in the next annual trachoma-specific article published in the Weekly Epidemiological Record,^30^ and changes the trachoma endemicity status of the Member State in the Global Health Observatory^120^ to “validated as having eliminated trachoma as a public health problem”.^30^

### Progress Towards Elimination

The number of people at risk of trachoma has fallen by 93%, from 1.5 billion in 2002 to 103.2 million in April 2024, and the number of people with TT has fallen by 80% in the same time-period, from 7·6 million to 1·5 million.^30^ As of March 2025, 21 countries have been validated by WHO as having eliminated trachoma as a public health problem: Benin, Cambodia, China, Gambia, Ghana, India, Iraq, Islamic Republic of Iran, Lao People’s Democratic Republic, Malawi, Mali, Mexico, Morocco, Myanmar, Nepal, Oman, Pakistan, Saudi Arabia, Togo and Vanuatu and Viet Nam.^121^ Seven more countries claim to have met the prevalence criteria for elimination: Botswana, Burundi, Guatemala, Mauritania, Namibia, Papua New Guinea, and Tunisia.^30^ Although models predict that the majority of EUs will meet the elimination targets by 2030, a small proportion may require additional efforts if the 2030 target is to be met.^122,123^

### Integration & Mainstreaming

In the 2021–2030 NTD road map, the NTD community committed to a shift from siloed disease-specific programmes to holistic, cross-cutting approaches, including integration across NTDs and beyond, and mainstreaming into the national health system.^1^ Integration and mainstreaming are crucial to a successful and sustainable trachoma elimination programme. In trachoma elimination, integration could include the joint delivery of TT surgical activities with other health care activities, azithromycin MDA with other NTDs’ MDA, F & E interventions co-delivered with other sectors, and integrated monitoring, evaluation and reporting to maximise efficiency. Mainstreaming trachoma initiatives ensures trachoma control and surveillance are embedded in national policies, frameworks, and systems for sustainable, efficient prevention and control.^1^ It enables patients to access all treatment, care and support that they need, even in the post-elimination era.

Weak health systems and inadequate capacity pose challenges to successful transition and sustainability of interventions and surveillance.^124^ Countries are expected to strengthen all critical components of their health system supported by context-specific evidence prior to embarking into mainstreaming efforts.^1,124^ The F & E components of the SAFE strategy depend on the education, water resources and environmental sectors. However, these sectors often operate independently from health, necessitating a coordinated approach to planning, budgeting and implementation. The WHO WASH-NTD guide and toolkit offers guidance on how to effectively engage and work collaboratively with these adjacent sectors.^125^

### Challenges

#### Persistent and recrudescent active trachoma

Persistent and recrudescent active trachoma coupled with insecurity to operate in several trachoma-endemic settings presently challenge the success of the entire 2030 global elimination target.^126^ There is growing evidence that in some hyperendemic populations, the elimination threshold of <5% TF prevalence in children aged 1–9 years (TF1-9) is not met despite years of implementation of recommended interventions.^127,128^ In some hyperendemic areas, the currently recommended single annual community wide antibiotic schedule appears insufficient to reliably achieve long-term control, with re-emergence of disease being typical.^129,130^ As of June 2024, 253 EUs (16·8% of all ever-endemic districts worldwide) had persistent trachoma, defined as undergoing two or more trachoma impact surveys with TF1-9 never being below 5%. Another 174 districts (10·4% of all ever-endemic districts) had recrudescent trachoma, defined by at least one trachoma surveillance survey having returned a TF1-9 ≥5% (with current prevalence remaining ≥5%).

The biological or methodological reasons for persistence and recrudescence are not clearly understood. The most consistent predictor has been high prevalence of TF at baseline. Addressing persistent and recrudescent trachoma requires a context specific, multifaceted, innovative approach supported by evidence. In WHO’s 2021 informal consultation on end-game challenges for trachoma elimination, it was recognised that the evidence base for optimal management of persistent and recrudescent trachoma is weak, and that, as a consequence, tailored management guided by expert opinion is likely to be the most appropriate current course of action.^126^ Among the suggested tailored management options that could be adopted by national programmes are: a) more MDA rounds and extended periods of F and E intervention before re-survey; b) more frequent than annual MDA rounds, with the possibility of additional MDA rounds only to demographic subgroups likely to have the highest prevalence of conjunctival *Ct* infection; and c) more intensive F and E interventions.^126^

#### Postoperative TT

PTT is the most important and most frequently observed measure of undesirable surgical outcomes. PTT incidence varies by the type of surgical procedure and follow-up time. Studies have reported a PTT cumulative incidence of 2% at 6 weeks and 60% at 3 years after surgery, with an average of around 20% by 1 year.^65,68,131–133^ There is a consistent pattern to PTT: an initially high rate during the first six months, followed by a slower rate after six months. A study in Ethiopia reported a PTT rate of 3·5% per month during the first six months, dropping to 0·8% per month between six and 24 months (RR 0·24, p=0·0001).^134^

PTT is a significant challenge in preventing blindness from trachoma in several ways. First, PTT sustains eye pain and increases the risk of CO and vision loss, negatively impacting quality of life and mental health. Second, PTT deters other patients from seeking out or accepting TT surgery, hampering surgical uptake. Third, management of PTT requires a higher level of expertise than the initial operation, absorbing significant resources within trachoma programmes that are often already overstretched.

There is a need to collect data on the proportion of TT patients who are effectively managed and not requiring further active management after their primary operations. WHO recommends that programmes should aim to reduce cumulative PTT incidence to below 10% at 6 months for cases with minor preoperative TT (≤5 eyelashes) and below 20% for cases with major postoperative TT (>5 eyelashes).^133^ Use of updated resources and tools, including surgical training manuals, and surgical simulators such as Head Start,^135^ to standardise and improve surgeon performance, and periodic review of surgeons’ skill through analysis of surgical outcomes, could help to enhance our collective ability to improve surgical quality.^64^

#### Sustaining Elimination & Post-validation Surveillance

Part of the trachoma elimination validation dossier is outlining national plans for post-validation surveillance, including provision of surgical services for incident TT cases, and continued health ministry engagement with other government ministries for the provision of WASH services. Countries must also demonstrate that they are committed to continued surveillance, so that any recrudescent disease can be detected, which should then be reported to WHO’s Global NTD Programme and noted in the Global Health Observatory and WHO’s Weekly Epidemiological Record.^136^ WHO does not specify how countries should conduct post-validation surveillance, as the appropriate methodology will be country- and context-specific.

As such, different countries have employed different approaches.^137,138^ Most countries are employing passive surveillance approaches, with TF and TT identification and reporting integrated into national integrated surveillance systems,^139,140^ with training to ensure accurate detection of cases, and referral systems for the appropriate management of individuals with TT. Some countries are also using active surveillance strategies, such as prevalence surveys and sentinel site monitoring. Furthermore, the use of complementary indicators to detect evidence of current and past *C. trachomatis* infection is being used, for example in Ghana and Morocco.^140,141^ Through these efforts, countries are able to detect indications of disease recrudescence, identify the target areas and population groups involved, plan effective delivery of the necessary interventions, and continue to advocate for resources.

### Case Study

Nepal and Ethiopia have had contrasting experiences in trying to eliminate trachoma. Here, we describe some of their successes and challenges to provide locally-grounded examples of overall global progress.

#### Nepal

In 1981, trachoma was the second commonest cause of preventable blindness nationally, and highly endemic in many parts of the country, with prevalence estimates of active trachoma as high as 23% in several districts. The National Trachoma Program (NTP) was launched in 2002 and conducted population-based trachoma prevalence surveys throughout the country. These surveys identified trachoma as a public health problem in 20 of 75 districts. The NTP adopted an integrated approach to trachoma elimination, with Nepal Netra Jyoti Sangh (NNJS), a national NGO leading the surgical component, the Ministry of Health and Population (MoHP) overseeing antibiotic MDA, and water, sanitation and education partners delivering the “F” and “E” components of SAFE. On 22 November 2018, WHO congratulated Nepal, the first country in WHO’s South-East Asia Region, for eliminating trachoma as a public health problem.

Elimination of trachoma in Nepal had many challenges, including difficult geography, severely limited financial resources and political upheaval. However, the country overcame each issue by applying an integrated and holistic approach, efficient administrative coordination between stakeholders, strong political support, social mobilisation and ownership of the programme by NNJS. Nepal has demonstrated that carefully planned strategies, implemented with the involvement of all technical and social stakeholders, can lead to successful elimination of trachoma.

#### Ethiopia

**Figure 5:**
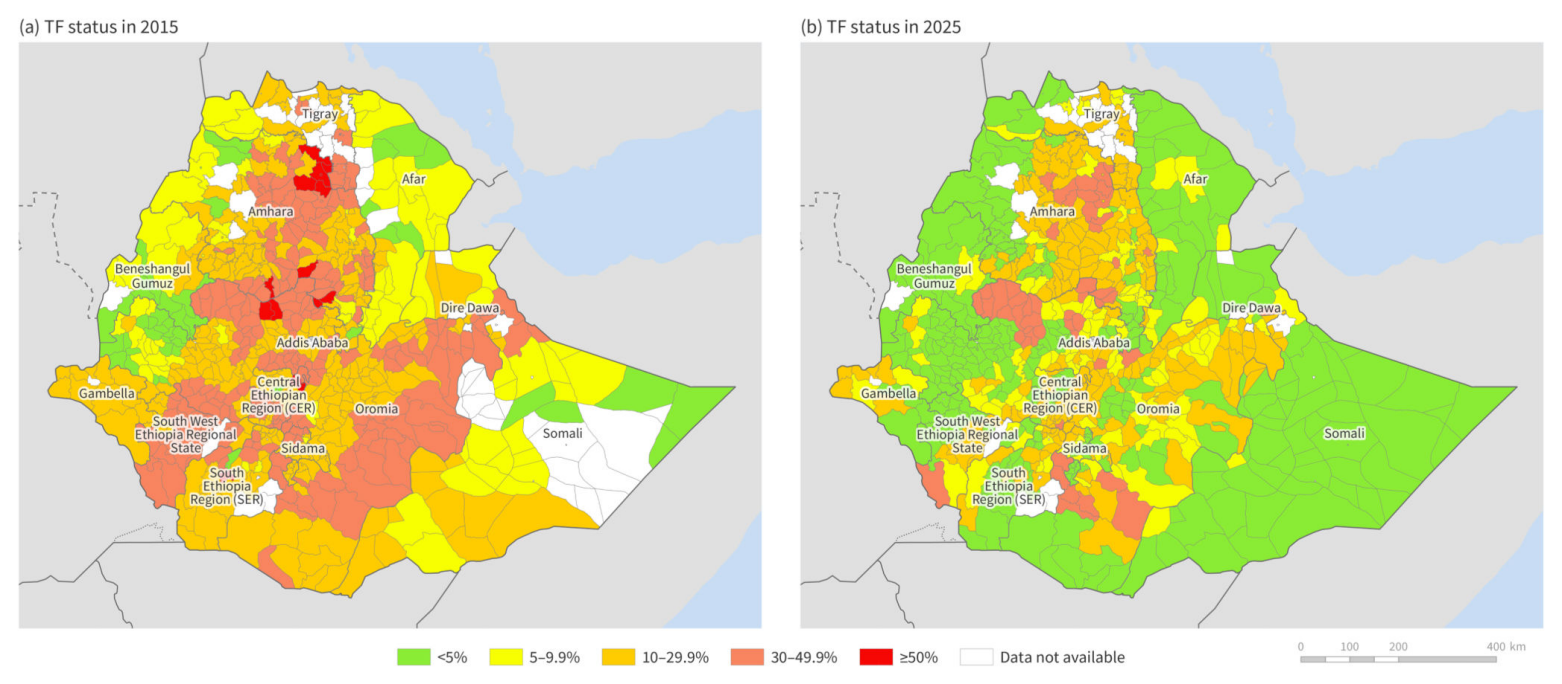
Ethiopia Trachoma Elimination Progress. **Adapted from the Trachoma Atlas February 2025**, https://www.trachomaatlas.org. The boundaries and names shown, and the designations used on this map do not imply the expression of any opinion whatsoever on the part of the authors, or the institutions with which they are affiliated, concerning the legal status of any country, territory, city or area or of its authorities, or concerning the delimitation of its frontiers or boundaries

In Ethiopia, the SAFE strategy has been implemented as a national programme since 2003. More than 1·7 million TT operations have been performed, ~500 million doses of azithromycin have been distributed, and millions of pit latrines built. Thanks to these interventions, significant progress has been made. The mean EU-level TF prevalence fell from 26·2% in 2015 to 9·3% in 2023, a 64·5% reduction. The mean EU-level prevalence of TT fell from 2·07% in 2003 to 0·80% in 2023, a 61% reduction. As of 2023, among 850 districts known to have had a TT prevalence above the elimination threshold at baseline, 132 (15·5%) were below the elimination threshold. Similarly, among 886 districts with a baseline TF prevalence above the elimination threshold at baseline, 320 (36·1%) were below the elimination threshold, Figure 5.

Despite this encouraging progress, after two decades of SAFE strategy implementation, including up to 15 rounds of azithromycin MDA for some populations, trachoma persists in some districts, with a prevalence far above the WHO elimination threshold.^142^ As of June 2024, Ethiopia has about 84% and 62% of all districts worldwide known to have persistent and recrudescent active trachoma, respectively. There is consensus among stakeholders in Ethiopia that continuing the same pattern of SAFE strategy implementation (“business as usual”) is unlikely to lead to elimination by 2030, and novel, potentially more effective approaches need to be developed, tested, and rolled out. The Ethiopian Ministry of Health, in addition to strengthening the F & E interventions, is embarking on a trial of doubling the frequency of azithromycin MDA in children, to accelerate trachoma elimination in districts with persistent disease. These efforts need to be supported by high MDA coverage across communities, and behavioural change interventions that promote consistent personal and environmental hygiene practices, along with a monitoring system.

## Conclusions

The SAFE strategy has proven effective in eliminating trachoma as a public health problem in several settings. Many countries are expected to be validated as having eliminated trachoma as a public health problem by 2030. However, some countries with hyperendemic trachoma may struggle to achieve elimination by 2030 due to persistent and recrudescent active trachoma and insecurity. Although there is no certainty as to why trachoma persists or recrudesces, in hyperendemic areas, single annual community-wide antibiotic MDA schedules appear insufficient to reliably achieve sustained elimination. In such settings, understanding the context-specific reasons for persistent and recrudescent trachoma should be a key area of research to develop tailored interventions. The use of alternative indicators, such as prevalence of Ct infection and antibodies in children at the community level, may help inform programmatic decisions, particularly in settings with persistent or recurrent active trachoma. The role of other factors, such as *Ct* strain variability, antibiotic resistance, different host immune responses, suboptimal MDA coverage, micronutrient deficiency or abundance, and inconsistent personal and environmental hygiene need to be studied as potential contributors. The added benefits of additional rounds of MDA, more frequent than annual MDA, and more intensive delivery of F and E interventions should be tested in implementation research. The strong immune responses observed in the Phase I chlamydia vaccine trials are promising, highlighting the need for further research to optimize dosage and delivery methods. Mainstreaming interventions into the primary health care system, maximising efficiency through integrated approaches, and collaboration with other sectors to implement F & E interventions should be considered. Further work is needed on strategies to maximise not only the proportion of TT cases managed and known to the health system but also effectively managed and not requiring further investment in the post-elimination era.

## Figures and Tables

**Table 1:** World Health Organization Follicles-Papillae-Cicatricae (FPC) grading system ^46,47^

	
Grade	Description
Upper Tarsal Follicles (F)	
F 0	No follicles.
F 1	Follicles present, but no more than 5 in zones 2 and 3 together (see Figure 4)
F 2	More than 5 follicles in zones 2 and 3 together, but less than 5 in zone 3.
F 3	Five or more follicles in each of the three zones.

Upper tarsal papillary hypertrophy and diffuse inflammation (P)	
P 0	Absent: normal appearance
P 1	Minimal: individual vascular tufts (papillae) prominent, but deep subconjunctival vessels on the tarsus not obscured.
P 2	Moderate: more prominent papillae, and normal vessels appear hazy, even when seen by the naked eye.
P 3	Pronounced: conjunctiva thickened and opaque, normal vessels on the tarsus are hidden over more than half of the surface.

Conjunctival scaring (C)	
C 0	No scarring on the conjunctiva
C 1	Mild: fine scattered scars on the upper tarsal conjunctiva, or scars on other parts of the conjunctiva.
C 2	Moderate: more severe scarring but without shortening or distortion of the upper tarsus.
C 3	Severe: scarring with distortion of the upper tarsus.

Trichiasis and/or entropion (T/E)	
T/E 0	No trichiasis and/or entropion.
T/E 1	Lashes deviated towards the eye, but not touching the globe.
T/E 2	Lashes touching the globe but not rubbing the cornea.
T/E 3	Lashes constantly rubbing the cornea.

Corneal scarring (CC)	
CC 0	Absent
CC 1	Minimal scarring or opacity but not involving the visual axis, and with clear central cornea.
CC 2	Moderate scarring or opacity involving the visual axis, with the papillary margin visible through the opacity.
CC 3	Severe central scarring or opacity with the papillary margin not visible through the opacity.

## References

[1] World Health Organization (2020). Ending the neglect to attain the sustainable development goals: a road map for neglected tropical diseases 2021–2030.

[2] Pascolini D, Mariotti SP (2012). Global estimates of visual impairment: 2010. British Journal of Ophthalmology.

[3] Burton MJ, Mabey DCW (2009). The Global Burden of Trachoma: A Review. PLoS Negl Trop Dis.

[4] Frick KD, Melia BM, Buhrmann RR, West SK (2001). Trichiasis and disability in a trachoma-endemic area of Tanzania. Archives of ophthalmology.

[5] Palmer SL, Winskell K, Patterson AE (2014). ‘A living death’: a qualitative assessment of quality of life among women with trichiasis in rural Niger. International health.

[6] Woreta TA, Munoz BE, Gower EW, Alemayehu W, West SK (2009). Effect of trichiasis surgery on visual acuity outcomes in Ethiopia. Archives of ophthalmology (Chicago, Ill : 1960).

[7] Wolle MA, Cassard SD, Gower EW (2011). Impact of Trichiasis surgery on physical functioning in Ethiopian patients: STAR trial. Am J Ophthalmol.

[8] Frick KD, Hanson CL, Jacobson GA (2003). Global burden of trachoma and economics of the disease. The American journal of tropical medicine and hygiene.

[9] Solomon AW, Burton MJ, Gower EW (2022). Trachoma. Nat Rev Dis Primers.

[10] Emerson PM, Cairncross S, Bailey RL, Mabey DCW (2000). Review of the evidence base for the ‘F’ and ‘E’ components of the SAFE strategy for trachoma control. Tropical Medicine & International Health.

[11] Wright HR, Turner A, Taylor HR (2007). Trachoma and poverty: unnecessary blindness further disadvantages the poorest people in the poorest countries. Clinical & experimental optometry : journal of the Australian Optometrical Association.

[12] Taylor HR, Burton MJ, Haddad D, West S, Wright H (2014). Trachoma. The Lancet.

[13] Schachter J, Dawson C (1978). Human Chlamydial Infections.

[14] Solomon AW, Kello AB, Bangert M (2020). The simplified trachoma grading system, amended. Bull World Health Organ.

[15] Newhall WJt, Terho P, Wilde CE, Batteiger BE, Jones RB (1986). Serovar determination of Chlamydia trachomatis isolates by using type-specific monoclonal antibodies. J Clin Microbiol.

[16] Frost EH, Deslandes S, Veilleux S, Bourgaux-Ramoisy D (1991). Typing Chlamydia trachomatis by detection of restriction fragment length polymorphism in the gene encoding the major outer membrane protein. J Infect Dis.

[17] Hadfield J, Harris SR, Seth-Smith HMB (2017). Comprehensive global genome dynamics of Chlamydia trachomatis show ancient diversification followed by contemporary mixing and recent lineage expansion. Genome Res.

[18] Stephens RS, Kalman S, Lammel C (1998). Genome sequence of an obligate intracellular pathogen of humans: Chlamydia trachomatis. Science.

[19] Mabey DCW, Solomon AW, Foster A (2003). Trachoma. The Lancet.

[20] Burton MJ, Holland MJ, Faal N (2003). Which members of a community need antibiotics to control trachoma? Conjunctival Chlamydia trachomatis infection load in Gambian villages. Invest Ophthalmol Vis Sci.

[21] Ramadhani AM, Derrick T, Macleod D, Holland MJ, Burton MJ (2016). The Relationship between Active Trachoma and Ocular Chlamydia trachomatis Infection before and after Mass Antibiotic Treatment. PLOS Neglected Tropical Diseases.

[22] Bailey R, Duong T, Carpenter R, Whittle H, Mabey D (1999). The duration of human ocular Chlamydia trachomatis infection is age dependent. Epidemiology and Infection.

[23] Derrick T, Ramadhani AM, Macleod D (2020). Immunopathogenesis of Progressive Scarring Trachoma: Results of a 4-Year Longitudinal Study in Tanzanian Children. Infection and Immunity.

[24] Ramadhani AM, Derrick T, Macleod D (2019). Progression of scarring trachoma in Tanzanian children: A four-year cohort study. PLoS Negl Trop Dis.

[25] Burton MJ, Rajak SN, Hu VH (2015). Pathogenesis of Progressive Scarring Trachoma in Ethiopia and Tanzania and Its Implications for Disease Control: Two Cohort Studies. PLoS Negl Trop Dis.

[26] Wolle MA, Muñoz B, Mkocha H, West SK (2009). Age, Sex, and Cohort Effects in a Longitudinal Study of Trachomatous Scarring. Investigative ophthalmology & visual science.

[27] Wolle MA, Muñoz BE, Mkocha H, West SK Constant Ocular Infection with Chlamydia trachomatis Predicts Risk of Scarring in Children in Tanzania. Ophthalmology.

[28] West SK, Munoz B, Mkocha H, Hsieh YH, Lynch MC (2001). Progression of active trachoma to scarring in a cohort of Tanzanian children. Ophthalmic Epidemiol.

[29] Gambhir M, Basáñez M-G, Burton MJ (2009). The Development of an Age-Structured Model for Trachoma Transmission Dynamics, Pathogenesis and Control. PLOS Neglected Tropical Diseases.

[30] World Health Organization Weekly Epidemiological Record (2024). WHO Alliance for the Global Elimination of Trachoma: progress report on elimination of trachoma, 2023.

[31] Gower EW, Solomon AW, Burton MJ (2006). Chlamydial positivity of nasal discharge at baseline is associated with ocular chlamydial positivity 2 months following azithromycin treatment. Invest Ophthalmol Vis Sci.

[32] Last A, Versteeg B, Abdurahman Shafi (2020). Detecting extra-ocular Chlamydia trachomatis in a trachoma-endemic community in Ethiopia: Identifying potential routes of transmission. PLOS Neglected Tropical Diseases.

[33] Burgert-Brucker CR, Adams MW, Mingkwan P (2022). Community-level trachoma ecological associations and the use of geospatial analysis methods: A systematic review. PLoS Negl Trop Dis.

[34] Nash SD, Sata E, Chernet A (2024). The Epidemiology of Ocular Chlamydia trachomatis Infection within Districts Persistently Endemic for Trachoma in Amhara, Ethiopia. The American journal of tropical medicine and hygiene.

[35] Bailey R, Osmond C, Mabey DC, Whittle HC, Ward ME (1989). Analysis of the household distribution of trachoma in a Gambian village using a Monte Carlo simulation procedure. Int J Epidemiol.

[36] Garn JV, Boisson S, Willis R (2018). Sanitation and water supply coverage thresholds associated with active trachoma: Modeling cross-sectional data from 13 countries. PLOS Neglected Tropical Diseases.

[37] Sullivan KM, Harding-Esch EM, Keil AP (2023). Exploring water, sanitation, and hygiene coverage targets for reaching and sustaining trachoma elimination: G-computation analysis. PLOS Neglected Tropical Diseases.

[38] West SK, Congdon N, Katala S, Mele L (1991). FAcial cleanliness and risk of trachoma in families. Archives of Ophthalmology.

[39] Shafi Abdurahman O, Last A, Macleod D (2023). Trachoma risk factors in Oromia Region, Ethiopia. PLoS Negl Trop Dis.

[40] Last AR, Burr SE, Weiss HA (2014). Risk Factors for Active Trachoma and Ocular Chlamydia trachomatis Infection in Treatment-Naïve Trachoma-Hyperendemic Communities of the Bijagós Archipelago, Guinea Bissau. PLoS Negl Trop Dis.

[41] Emerson PM, Bailey RL, Mahdi OS, Walraven GE, Lindsay SW (2000). Transmission ecology of the fly Musca sorbens, a putative vector of trachoma. Trans R Soc Trop Med Hyg.

[42] Emerson PM, Lindsay SW, Alexander N (2004). Role of flies and provision of latrines in trachoma control: cluster-randomised controlled trial. The Lancet.

[43] Schémann J-F, Sacko D, Malvy D (2002). Risk factors for trachoma in Mali. International Journal of Epidemiology.

[44] Courtright P, West SK (2004). Contribution of sex-linked biology and gender roles to disparities with trachoma. Emerg Infect Dis.

[45] Solomon AW, Peeling RW, Foster A, Mabey DC (2004). Diagnosis and assessment of trachoma. Clin Microbiol Rev.

[46] Dawson CR, Jones BR, Darougar S (1975). Blinding and non-blinding trachoma: assessment of intensity of upper tarsal inflammatory disease and disabling lesions. Bulletin of the World Health Organization.

[47] Dawson CR, Jones BR, Tarizzo ML, World Health Organization (1981). Guide to trachoma control in programmes for the prevention of blindness.

[48] Thylefors B, Dawson CR, Jones BR, West S, Taylor HR (1987). A simple system for the assessment of trachoma and its complications. Bulletin of the World Health Organization.

[49] Butcher R, Handley B, Garae M (2020). Ocular Chlamydia trachomatis infection, anti-Pgp3 antibodies and conjunctival scarring in Vanuatu and Tarawa, Kiribati before antibiotic treatment for trachoma. J Infect.

[50] Atekem K, Harding-Esch EM, Martin DL (2023). High prevalence of trachomatous inflammation-follicular with no trachomatous trichiasis: can alternative indicators explain the epidemiology of trachoma in Côte d’Ivoire?. Int Health.

[51] Sata E, Seife F, Ayele Z (2024). Wait and watch: A trachoma surveillance strategy from Amhara region, Ethiopia. PLoS Negl Trop Dis.

[52] Solomon AW, Holland MJ, Burton MJ (2003). Strategies for control of trachoma: observational study with quantitative PCR. Lancet.

[53] Jalal H, Stephen H, Curran MD, Burton J, Bradley M, Carne C (2006). Development and validation of a rotor-gene real-time PCR assay for detection, identification, and quantification of Chlamydia trachomatis in a single reaction. J Clin Microbiol.

[54] Roberts CH, Last A, Molina-Gonzalez S (2013). Development and evaluation of a next-generation digital PCR diagnostic assay for ocular Chlamydia trachomatis infections. J Clin Microbiol.

[55] Versteeg B, Vasileva H, Houghton J (2020). Viability PCR shows that non-ocular surfaces could contribute to transmission of Chlamydia trachomatis infection in trachoma. PLoS Negl Trop Dis.

[56] Solomon AW, Mohammed Z, Massae PA (2005). Impact of mass distribution of azithromycin on the antibiotic susceptibilities of ocular Chlamydia trachomatis. Antimicrob Agents Chemother.

[57] Pickering H, Chernet A, Sata E (2020). Genomics of Ocular Chlamydia trachomatis after 5 years of SAFE interventions for trachoma in Amhara, Ethiopia. J Infect Dis.

[58] Martin DL, Saboya-Diaz MI, Abashawl A (2020). The use of serology for trachoma surveillance: Current status and priorities for future investigation. PLoS Negl Trop Dis.

[59] Tedijanto C, Solomon AW, Martin DL (2023). Monitoring transmission intensity of trachoma with serology. Nat Commun.

[60] Francis V, Turner V (1995). Achieving community support for trachoma control. A guide for district health work.

[61] Abraham S, Juel HB, Bang P (2019). Safety and immunogenicity of the chlamydia vaccine candidate CTH522 adjuvanted with CAF01 liposomes or aluminium hydroxide: a first-in-human, randomised, double-blind, placebo-controlled, phase 1 trial. The Lancet Infectious Diseases.

[62] Pollock KM, Borges ÁH, Cheeseman HM (2024). An investigation of trachoma vaccine regimens by the chlamydia vaccine CTH522 administered with cationic liposomes in healthy adults (CHLM-02): a phase 1, double-blind trial. The Lancet Infectious Diseases.

[63] Burton MJ, Bowman RJC, Faal H (2005). Long term outcome of trichiasis surgery in the Gambia. British Journal of Ophthalmology.

[64] World Health Organization (2024). Trichiasis surgery for trachoma.

[65] Rajak SN, Collin JR, Burton MJ (2012). Trachomatous trichiasis and its management in endemic countries. Survey of ophthalmology.

[66] Reacher MH, Taylor HR (1990). The management of trachomatous trichiasis. Revue internationale du trachome et de pathologie oculaire tropicale et subtropicale et de sante publique : organe de la Ligue contre le trachome avec la collaboration de l’International Organization.

[67] Yorston D, Mabey D, Hatt S, Burton M (2006). Interventions for trachoma trichiasis. Cochrane Database of Systematic Reviews.

[68] Burton MJ, Habtamu E, Ho D, Gower EW (2015). Interventions for trachoma trichiasis. Cochrane Database of Systematic Reviews.

[69] World Health Organization Alliance for the Global Elimination of Trachoma by 2020 (2015). Second Global Scientific Meeting on Trachomatous Trichiasis.

[70] Habtamu E, Wondie T, Aweke S (2016). Posterior lamellar versus bilamellar tarsal rotation surgery for trachomatous trichiasis in Ethiopia: a randomised controlled trial. The Lancet Global Health.

[71] Habtamu E, Wondie T, Tadesse Z (2019). Posterior lamellar versus bilamellar tarsal rotation surgery for trachomatous trichiasis: Long-term outcomes from a randomised controlled trial. EClinicalMedicine.

[72] Burton MJ, Bowman RJ, Faal H (2006). The long-term natural history of trachomatous trichiasis in the Gambia. Invest Ophthalmol Vis Sci.

[73] Rajak SN, Habtamu E, Weiss HA (2012). Epilation for Trachomatous Trichiasis and the Risk of Corneal Opacification. Ophthalmology.

[74] Habtamu E, Wondie T, Gobezie W (2020). Effect of repeated epilation for minor trachomatous trichiasis on lash burden, phenotype and surgical management willingness: A cohort study. PLOS Neglected Tropical Diseases.

[75] Rajak SN, Habtamu E, Weiss HA (2011). Surgery Versus Epilation for the Treatment of Minor Trichiasis in Ethiopia: A Randomised Controlled Noninferiority Trial. PLoS Med.

[76] Habtamu E, Rajak SN, Tadesse Z (2015). Epilation for Minor Trachomatous Trichiasis: Four-Year Results of a Randomised Controlled Trial. PLoS Negl Trop Dis.

[77] Ridgway G (1997). Treatment of chlamydial genital infection. J Antimicrob Chemother.

[78] Bailey RL, Arullendran P, Whittle HC, Mabey DC (1993). Randomised controlled trial of single-dose azithromycin in treatment of trachoma. Lancet.

[79] Dawson CR, Schachter J, Sallam S, Sheta A, Rubinstein RA, Washton H (1997). A comparison of oral azithromycin with topical oxytetracycline/polymyxin for the treatment of trachoma in children. Clinical Infectious Diseases.

[80] Tabbara KF, Abu-el-Asrar A, al-Omar O, Choudhury AH, al-Faisal Z (1996). Single-dose azithromycin in the treatment of trachoma. A randomized, controlled study. Ophthalmology.

[81] Schachter J, West SK, Mabey D (1999). Azithromycin in control of trachoma. Lancet.

[82] Solomon AW, World Health O, London School of H, Tropical M, International Trachoma I (2006). Trachoma control : a guide for programme managers.

[83] World Health O (2010). Report of the 3rd Global scientific meeting on trachoma: Baltimore, USA, 19-20 July, 2010.

[84] Chidambaram JD, Alemayehu W, Melese M (2006). Effect of a single mass antibiotic distribution on the prevalence of infectious trachoma. JAMA.

[85] Solomon AW, Holland MJ, Alexander ND (2004). Mass treatment with single-dose azithromycin for trachoma. N Engl J Med.

[86] Lietman T, Porco T, Dawson C, Blower S (1999). Global elimination of trachoma: how frequently should we administer mass chemotherapy?. Nature Medicine.

[87] Melese M, Chidambaram JD, Alemayehu W (2004). Feasibility of eliminating ocular Chlamydia trachomatis with repeat mass antibiotic treatments. JAMA.

[88] Gill DA, Lakew T, Alemayehu W (2008). Complete Elimination Is a Difficult Goal for Trachoma Programs in Severely Affected Communities. Clin Infect Dis.

[89] Biebesheimer JB, House J, Hong KC (2009). Complete Local Elimination of Infectious Trachoma from Severely Affected Communities after Six Biannual Mass Azithromycin Distributions. Ophthalmology.

[90] Melese M, Alemayehu W, Lakew T (2008). Comparison of annual and biannual mass antibiotic administration for elimination of infectious trachoma. Jama.

[91] Gebre T, Ayele B, Zerihun M (2012). Comparison of annual versus twice-yearly mass azithromycin treatment for hyperendemic trachoma in Ethiopia: a cluster-randomised trial. Lancet.

[92] House JI, Ayele B, Porco TC (2009). Assessment of herd protection against trachoma due to repeated mass antibiotic distributions: a cluster-randomised trial. Lancet.

[93] Lietman TM, Ayele B, Gebre T (2020). Frequency of Mass Azithromycin Distribution for Ocular Chlamydia in a Trachoma Endemic Region of Ethiopia: A Cluster Randomized Trial. Am J Ophthalmol.

[94] O’Brien KS, Emerson P, Hooper PJ (2019). Antimicrobial resistance following mass azithromycin distribution for trachoma: a systematic review. The Lancet Infectious Diseases.

[95] Evans JR, Solomon AW, Kumar R (2019). Antibiotics for trachoma. Cochrane Database Syst Rev.

[96] Amza A, Kadri B, Nassirou B (2017). A Cluster-Randomized Trial to Assess the Efficacy of Targeting Trachoma Treatment to Children. Clinical infectious diseases : an official publication of the Infectious Diseases Society of America.

[97] Mahmud H, Haile BA, Tadesse Z (2023). Targeted Mass Azithromycin Distribution for Trachoma: A Community-Randomized Trial (TANA II). Clinical infectious diseases : an official publication of the Infectious Diseases Society of America.

[98] Robinson A, Bickford-Smith J, Abdurahman Shafi O (2021). Towards an odour-baited trap to control Musca sorbens, the putative vector of trachoma. Scientific Reports.

[99] Robinson A, Gomes LRO, Abdurahman OS (2022). Evaluation of the efficacy of insecticide-treated scarves to protect children from the trachoma vector Musca sorbens (Diptera: Muscidae): A phase II randomised controlled trial in Oromia, Ethiopia. EClinicalMedicine.

[100] Ejere HO, Alhassan MB, Rabiu M (2015). Face washing promotion for preventing active trachoma. Cochrane Database Syst Rev.

[101] Rabiu M, Alhassan MB, Ejere HO, Evans JR (2012). Environmental sanitary interventions for preventing active trachoma. Cochrane Database Syst Rev.

[102] Aragie S, Wittberg DM, Tadesse W (2022). Water, sanitation, and hygiene for control of trachoma in Ethiopia (WUHA): a two-arm, parallel-group, cluster-randomised trial. Lancet Glob Health.

[103] Delea MG, Solomon H, Solomon AW, Freeman MC (2018). Interventions to maximize facial cleanliness and achieve environmental improvement for trachoma elimination: A review of the grey literature. PLoS Negl Trop Dis.

[104] Freeman MC, Delea MG, Snyder JS (2022). The impact of a demand-side sanitation and hygiene promotion intervention on sustained behavior change and health in Amhara, Ethiopia: A cluster-randomized trial. PLOS Glob Public Health.

[105] Garn JV, Boisson S, Willis R (2018). Sanitation and water supply coverage thresholds associated with active trachoma: Modeling cross-sectional data from 13 countries. PLoS Negl Trop Dis.

[106] Stocks ME, Ogden S, Haddad D, Addiss DG, McGuire C, Freeman MC (2014). Effect of water, sanitation, and hygiene on the prevention of trachoma: a systematic review and meta-analysis. PLoS Med.

[107] Sullivan KM, Harding-Esch EM, Keil AP (2023). Exploring water, sanitation, and hygiene coverage targets for reaching and sustaining trachoma elimination: G-computation analysis. PLoS Negl Trop Dis.

[108] Cairncross S (1999). Trachoma and water. Community Eye Health.

[109] Greenland K, Collin C, Sinba Etu E (2024). Comparison of metrics for assessing face washing behaviour for trachoma control. PLOS Neglected Tropical Diseases.

[110] Dodson S, Heggen A, Solomon AW, Sarah V, Woods G, Wohlgemuth L (2018). Behavioural change interventions for sustained trachoma elimination. Bull World Health Organ.

[111] Boisson S, Wohlgemuth L, Yajima A (2021). Building on a decade of progress in water, sanitation and hygiene to control, eliminate and eradicate neglected tropical diseases. Transactions of The Royal Society of Tropical Medicine and Hygiene.

[112] International Coalition for Trachoma Control (ICTC) (2019). Transition planning for trichiasis management services.

[113] Solomon AW, Pavluck AL, Courtright P (2015). The Global Trachoma Mapping Project: Methodology of a 34-Country Population-Based Study. Ophthalmic Epidemiol.

[114] Solomon AW, Willis R, Pavluck AL (2018). Quality Assurance and Quality Control in the Global Trachoma Mapping Project. The American journal of tropical medicine and hygiene.

[115] Harding-Esch EM, Bakhtiari A, Boyd S (2024). Tropical Data: supporting health ministries worldwide to conduct high-quality trachoma surveys. Int Health.

[116] Harding-Esch EM, Burgert-Brucker CR, Jimenez C (2023). Tropical Data: Approach and Methodology as Applied to Trachoma Prevalence Surveys. Ophthalmic Epidemiol.

[117] World Health Organization Strategic and Technical Advisory Group on Neglected Tropical Diseases (2014). Technical Consultation on Trachoma Surveillance. 2014 September 11−12; Task Force For Global HealtH, Decatur.

[118] World Health O (2017). Design and validation of a trachomatous trichiasis-only survey: Strategic and Technical Advisory Group for Neglected Tropical Diseases, working group on monitoring and evaluation.

[119] World Health Organization (2016). Validation of elimination of trachoma as a public health problem.

[120] World Health Organization (2024). The Global Health Observatory: Explore a world of health data.

[121] Jesudason T (2024). Pakistan, India, and Viet Nam eliminate trachoma. The Lancet Infectious Diseases.

[122] Blumberg S, Borlase A, Prada JM (2021). Implications of the COVID-19 pandemic in eliminating trachoma as a public health problem. Trans R Soc Trop Med Hyg.

[123] Srivathsan A, Abdou A, Al-Khatib T (2024). District-Level Forecast of Achieving Trachoma Elimination as a Public Health Problem By 2030: An Ensemble Modelling Approach. Clinical infectious diseases : an official publication of the Infectious Diseases Society of America.

[124] Dodson S, Gemechu A, Deneke YM, Bekere M, Belete S (2022). Understanding the facilitators and barriers to integrating trachoma interventions into routine health systems. Community Eye Health.

[125] World Health Organization (2023). WASH and health working together: a“ how-to” guide for neglected tropical disease programmes.

[126] World Health Organization (2021). Informal consultation on end-game challenges for trachoma elimination. Task Force for Global Health, Decatur, United States of America.

[127] Nash SD, Stewart AE, Zerihun M (2018). Ocular Chlamydia trachomatis infection under the surgery, antibiotics, facial cleanliness, and environmental improvement strategy in Amhara, Ethiopia, 2011–2015. Clinical Infectious Diseases.

[128] Stewart AE, Zerihun M, Gessese D (2019). Progress to eliminate trachoma as a public health problem in Amhara National Regional State, Ethiopia: results of 152 population-based surveys. The American journal of tropical medicine and hygiene.

[129] Lakew T, House J, Hong KC (2009). Reduction and return of infectious trachoma in severely affected communities in ethiopia. PLoSNeglTropDis.

[130] West SK, Munoz B, Mkocha H, Gaydos CA, Quinn TC (2011). Number of years of annual mass treatment with azithromycin needed to control trachoma in hyper-endemic communities in Tanzania. The Journal of infectious diseases.

[131] Gower EW, Merbs SL, Munoz BE (2011). Rates and risk factors for unfavorable outcomes 6 weeks after trichiasis surgery. Investigative ophthalmology & visual science.

[132] Khandekar R, Mohammed AJ, Courtright P (2001). Recurrence of trichiasis: a long-term follow-up study in the Sultanate of Oman. Ophthalmic Epidemiol.

[133] World Health Organization (2019). Report of the 4th global scientific meeting on trachoma: Geneva, 27–29 November 2018.

[134] Rajak SN, Habtamu E, Weiss HA (2011). Absorbable versus silk sutures for surgical treatment of trachomatous trichiasis in Ethiopia: a randomised controlled trial. PLoS medicine.

[135] Tadesse D, Montgomery I, Sankar G (2017). HEAD START - an innovative training approach for life-long learning. Community Eye Health.

[136] Control of Neglected Tropical Diseases (NTD), World Health Organization (2016). Validation of elimination of trachoma as a public health problem.

[137] Hammou J, Mohammadi F, Raisi AA, Harbi SA (2019). Sustaining trachoma elimination: lessons from North Africa and the Middle East. Community Eye Health.

[138] Quesada-Cubo V, Damián-González DC, Prado-Velasco FG (2022). The elimination of trachoma as a public health problem in Mexico: From national health priority to national success story. PLOS Neglected Tropical Diseases.

[139] Aboe A, Joof BM, Kanyi SK (2022). The Gambia has eliminated trachoma as a public health problem: Challenges and successes. PLOS Neglected Tropical Diseases.

[140] Senyonjo L, Addy J, Martin DL (2021). Surveillance for peri-elimination trachoma recrudescence: Exploratory studies in Ghana. PLoS Negl Trop Dis.

[141] Hammou J, Guagliardo SAJ, Obtel M (2022). Post-Validation Survey in Two Districts of Morocco after the Elimination of Trachoma as a Public Health Problem. The American journal of tropical medicine and hygiene.

[142] Sata E, Nute AW, Astale T (2021). Twelve-Year Longitudinal Trends in Trachoma Prevalence among Children Aged 1–9 Years in Amhara, Ethiopia, 2007–2019. The American journal of tropical medicine and hygiene.

